# Cardiovascular and Neurological Complications of COVID-19: A Narrative Review

**DOI:** 10.3390/jcm12082819

**Published:** 2023-04-12

**Authors:** Luma Ornelas Sousa Rêgo, Lara Landulfo Alves Braga, Gustavo Sampaio Vilas-Boas, Maiana Santos Oliveira Cardoso, Andre Rodrigues Duraes

**Affiliations:** 1 Bahiana School of Medicine and Public Health, BAHIANA/EBMSP, 275, Av. Dom João VI, Brotas, Salvador 40290-000, Brazil; lumarego19.2@bahiana.edu.br (L.O.S.R.);; 2 UNIFACS Medical School, Salvador University (UNIFACS), Salvador 40140-110, Brazil; 3 Bahiana Medical School of Federal University of Bahia, UFBA/FAMEB, PPGMS-EMBSP-Bahia Federal University, Salvador 40170-110, Brazil

**Keywords:** COVID-19, cardiovascular, cardiac, virus, neurological, complications

## Abstract

A novel coronavirus emerged in China in late 2019 as a disease named coronavirus disease 2019. This pathogen was initially identified as causing a respiratory syndrome, but later, it was found that COVID-19 could also affect other body systems, such as the neurological and cardiovascular systems. For didactic purposes, cardiovascular and neurological manifestations of SARS-CoV-2 have been classified in three different groups: acute complications, late complications, and post-vaccine complications. Therefore, the following study has the goal to summarize and disseminate the present knowledge about the cardiovascular and neurological manifestations of COVID-19 based on the latest and most up-to-date data available and, thus, promote more prepared medical care for these conditions as the medical team is updated. Based on what is brought on this revision and its understanding, the medical service becomes more aware of the causal relationship between some conditions and COVID-19 and can better prepare for the most prevalent conditions to associate and, consequently, to treat patients earlier. Therefore, there is a chance of better prognoses in this context and the need to increase the number of studies about complications related to SARS-CoV-2 infection for a better understanding of other associated conditions.

## 1. Introduction

In December 2019, an outbreak of a respiratory infection from unknown etiology happened in Wuhan, China [1]. Later, the responsible pathogen was identified as a new virus from the coronavirus family, severe acute respiratory syndrome coronavirus 2 (SARS-CoV-2), and the World Health Organization (WHO) named the disease as coronavirus disease 2019 (COVID-19) [2,3]. SARS-CoV-2 has proven to be highly transmissible, being capable of spreading to many countries beside China and, therefore, becoming a worldwide public health concern [2,3].

COVID-19 is able to infect people of all ages, although most cases seem to occur at the median age of 50 years [3,4]. However, the clinical presentation may vary among the different age groups. Children and young adults infected by SARS-CoV-2 often have mild symptoms, mild pneumonia, or asymptomatic infection [3]. In contrast, older adults, the elderly, and patients with comorbidities such as diabetes, obesity, chronic lung disease, or hypertension are at higher risk to develop a severe respiratory syndrome and systemic organ lesions, impacting on the need for hospitalization and mortality [3,4]. In addition, current studies have shown that several factors are correlated with the risk for the development of fibrosis and persistent functional deficit in COVID-19, such as severity of infection, need for ICU and mechanical ventilation, older age, higher body mass index, and smoking history [5].

The most common COVID-19 symptoms are fatigue, fever, and dry cough. In some cases, the first manifestations may be related in the gastrointestinal, neurological, or cardiovascular systems, such as diarrhea, anosmia, or palpitation [6,7,8]. In the study by Fritsche et al., conducted with 69 patients diagnosed with post-COVID syndrome, 22 had respiratory symptoms (e.g., dyspnea, cough, or hypoxemia), 13 had changes in the vascular system (orthostatic hypotension, and hypotension), 7 had neurological sequelae (sleep disorder, migraine, and pain), 6 had digestive symptoms (gastroesophageal reflux disease and irritable bowel syndrome), and 5 developed mental health problems (anxiety and depression) [9].

Even though it is not the majority of cases, some patients present cardiovascular complications of SARS-CoV-2 infection, such as arrhythmias, myocarditis, heart failure, bradycardia, thrombosis, and acute coronary syndromes [8]. In relation to neurological effects, the mechanisms by which SARS-CoV-2 damages the nervous system are still not clarified. In this sense, it is necessary to assess whether these manifestations are due to direct invasion, mediated by an indirect systemic inflammatory response or a combination of both [10].

The literature regarding COVID-19 is still being updated very fast, as new studies are published every day. Therefore, the goal of this study is to summarize and disseminate the present knowledge about the cardiovascular and neurological manifestations of COVID-19, based on the latest and most up-to-date data available and, thus, promote more prepared medical care for these conditions as the medical team is updated.

## 2. Methods

This study is a narrative review about neurological and cardiovascular manifestations resulting from infection by SARS-CoV-2, known as COVID-19, based on the indexed studies conducted. To this purpose, the authors followed two search strategies: one for the neurological complications and the other for the cardiovascular ones.

For the neurological complications search strategy, three databases widely used in the healthcare area were selected: SciELO (Scientific Electronic Library OnLine), MedLine (Medical Literature Analysis and Tetrietal System On-Line), and PubMed. Data were inspected based on the health sciences descriptors (DeCS) through the following descriptors: “Neurological Manifestations”, “Neurologic Deficits”, “Neurologic Symptoms”, “Neurologic Signs”, “COVID 19”, “SARS-CoV-2”, “Coronavirus Disease”, “SARS-CoV-2 Infection”, “Neurological Manifestations”, and “Neurological Deficits”, in their various combinations. At a certain point, the descriptors used were crossed with each other. 

The documents that constitute the context of the study were identified by the integrated search method in the search tool. The groups of descriptors were then used to identify the articles, considering, for this purpose, the period between December 2020 and November 2022, papers in English or Portuguese, and with free full text available. The databases were consulted between December 2022 and February 2023, and the research was conducted by two researchers independently and, then, compared to verify the similarity between the articles found. 

**Scheme 1:**
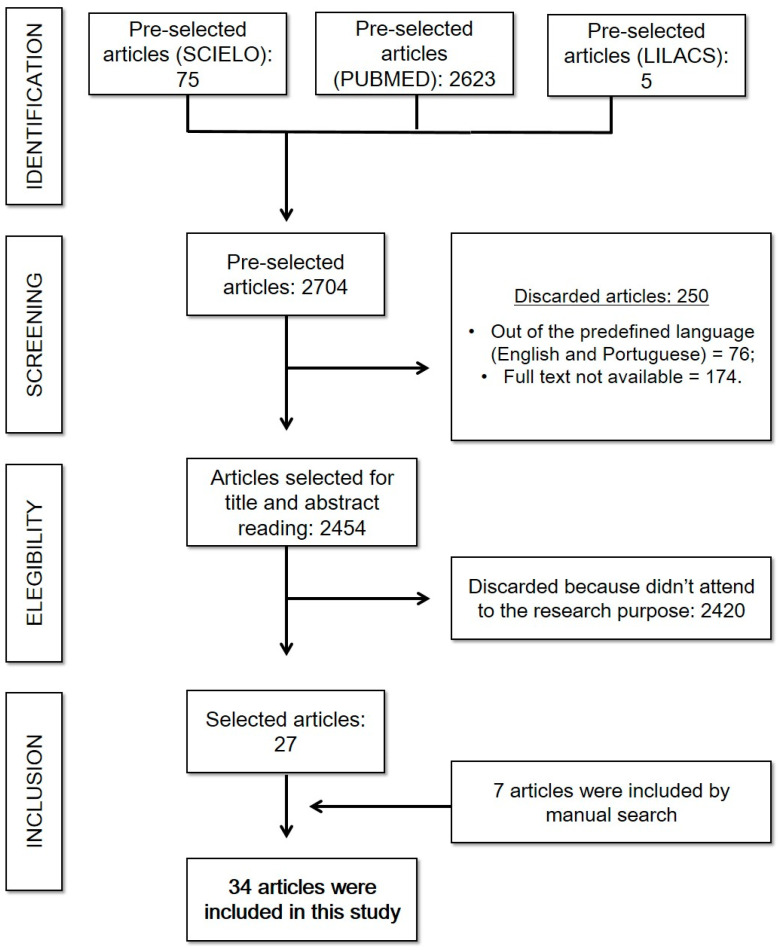
Neurological complications of COVID-19: search strategy. (Reference: elaborated by the authors).

Initially, still concerning the neurological search, 75 articles were found in SciELO, 5 articles in LILACS, and 2623 articles in PUBMED. However, of this total, only 1823 articles corresponded to the inclusion criteria, since 76 were not in the proposed languages and 174 were not available in full for free. Then, there was an analysis of the title and abstract to verify whether, in fact, they answer the guiding question and another 1791 were excluded for not meeting the objective—thus resulting in a set of 32 publications valid for this review, in addition to 7 articles added by manual search (some of them published in 2023). This search strategy is described in Scheme 1.

Conversely, for the cardiovascular search strategy, the authors used two databases: PubMed and Embase. The research was conducted from December 2022 to February 2023, based on the health sciences descriptors (DeCS) through the following descriptors: “Cardiovascular complications” AND “COVID-19”.

In the process, some filters were added as inclusion criteria: studies dating from 2020 to 2023, free full texts, clinical trials, systematic reviews, and observational studies.

**Scheme 2:**
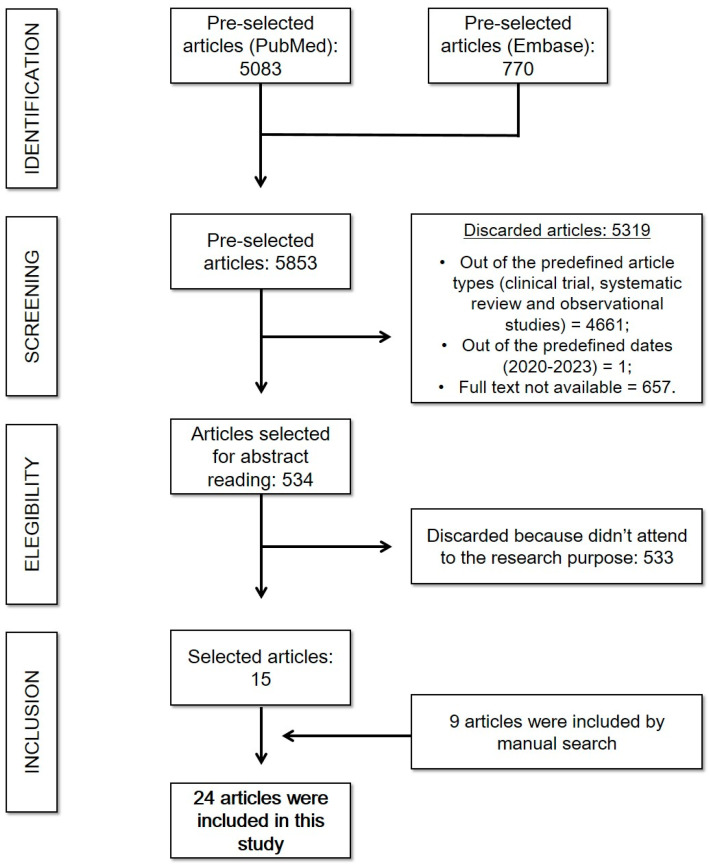
Cardiovascular complications of COVID-19: search strategy. (Reference: elaborated by the authors.).

Initially, 5083 articles were found on PubMed and 770 articles on Embase. Between those, only 534 matched the inclusion criteria, since 657 were not available in free full text mode, 1 did not correspond to the predefined publication dates, and 4661 studies did not match the study types included or were not related to the researched theme. Therefore, 534 articles were selected for abstract reading, of which only 15 corresponded to the research purpose and were included in the present study. In addition, 9 additional articles were included in the study through a manual search. This search strategy is described in Scheme 2.

For didactic purpose, this article is divided into acute, long-term, and post-vaccine complications.

## 3. Results

### 3.1. Pathophysiology and Clinical Features

**Figure 1:**
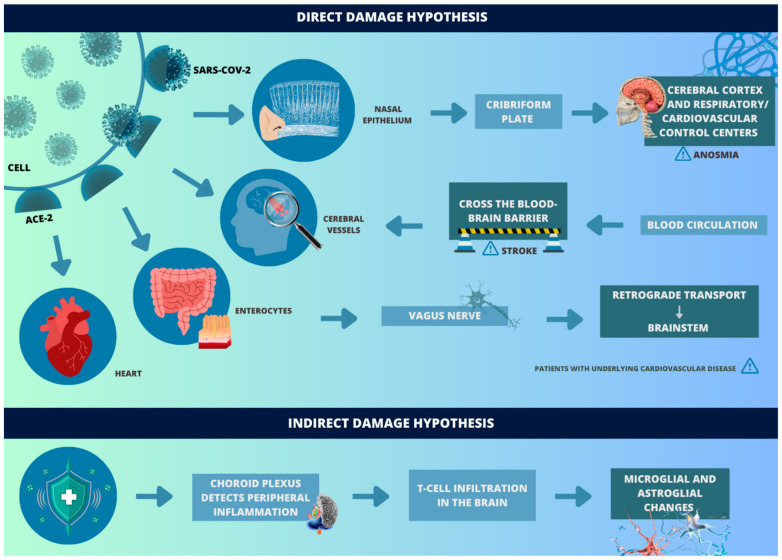
Hypothesis of direct and indirect damage by SARS-CoV-2. (REFERENCE: elaborated by the authors, based on Song J et al. [11], Hamming I et al. [12], Meinhardt J et al. [13], Achar A et al. [14], Awogbindin IO et al. [15], Yang AC et al. [16], Bourgonje AR et al. [17] and Chang WT et al. [18].

SARS-CoV-2 can enter the central nervous system (CNS) through different routes (Figure 1). For this to happen, angiotensin-converting enzyme 2 (ACE-2) acts as a receptor for the virus and enables binding to the surface of target cells through the spike glycoprotein (S) of the viral envelope, while serine transmembrane protease 2 (TMPRSS2), cell surface protein, cleaves glycoprotein S and, thus, allows the fusion of membranes and the endocytosis of the virus—which replicates and increases its invasive capacity [11]. In the nervous system, this receptor is mainly found in cerebral vessels, in the olfactory epithelium, and to a much lesser extent, in some parenchymal nerve cells [12].

The pathways by which SARS-CoV-2 interacts with the nervous system are, mainly, the pathway through the cribriform plate and the blood circulation. It is worth mentioning that, in both, the release of interferons type I occurs, which warns about the presence of the pathogen. When it occurs through the cribriform plate of the ethmoid bone, the virus ascends by retrograde axonal transport and, thus, passes from the nasal epithelium (which has ACE-2 receptors) to the cerebral cortex and respiratory/cardiovascular control centers in the brainstem [13]. In this way, the virus can adhere to motor proteins along the sensory and olfactory nerves, which is reinforced by the anosmia reported by the patients.

In the hematogenous route, after the respiratory tract infection, the virus is able to reach the CNS through the bloodstream. However, due to the unique configuration of the blood–brain barrier, which tightly regulates the molecule transport, SARS-CoV-2 cannot easily migrate from the capillaries of the systemic circulatory system to the brain through endothelial cells [14]. In this sense, transcellular migration, paracellular migration, and the “Trojan horse” strategy are the three main alternatives for the virus to cross this barrier [19]. In these mechanisms, the virus, respectively, invades the endothelial cells of the host to cross the blood–brain barrier, invades the junctions formed by the endothelial cells of the blood–brain barrier, and is phagocytosed by defense cells (such as neutrophils and macrophages) [19,20]. There are also studies that consider the possibility of another route: the gastrointestinal route, since ACE-2 is present in enterocytes. In this sense, the virus could reach terminal branches of the vagus nerve and, by retrograde transport, reach the brainstem—it is worth noting that both the nucleus of the solitary tract and the dorsal nucleus of the vagus nerve also express ACE-2 receptors [15].

The ACE-2 receptor, once bound to the virus, loses part of its function as a cerebrovascular protective factor, being able to generate high blood pressure and enhance the risk of intracerebral hemorrhages in patients with COVID-19. Furthermore, other neuronal complications, such as stroke, are favored by the disease’s systemic repercussions through a hypercoagulable state and cytokine storm [21]. Viral invasion of CNS endothelial cells results in neutrophil and macrophage activation, thrombin production, and complement activation, promoting thrombus formation. This phenomenon has been consistently observed in stroke events associated with COVID-19 [21].

However, current evidence suggests that direct viral damage is not the main mechanism for virus infection. In PCR tests collected from cerebrospinal fluid (CSF) in patients with COVID-19 and consequent neurological sequelae, viral genetic material is not usually present. In addition, patient autopsies demonstrate the absence of viral material in the frontal cortex and throughout the brain [22].

On the other hand, reinforcing the hypothesis of mostly indirect damage, cellular changes occur in the brain parenchyma, which suggest that the choroid plexus detects peripheral inflammation and relays it to the brain, where T cell infiltration is observed [16]. In these cases, the brain exhibits microglial and astroglial changes like those seen in various neurodegenerative diseases, as well as changes in synaptic transmission in neurons involved in cognitive function, which may explain the neurological involvement associated with COVID-19 [16]. In addition, a hypoxemic state, due to respiratory failure, damages the most sensitive brain regions, such as the hippocampus, involved in the formation of new memories and associated with learning and emotions, and the cerebellum, involved in motor coordination.

Oxidative stress and inflammation compromise the immunological response to COVID-19, leading to incomplete virus eradication. The viral residuals and antigen remnants lead to continued inflammatory response. In addition, a dysregulated immunologic response and virus-induced cytokine storm after the infection have been documented. Since the ACE-2 receptor is expressed in many organs, this prolonged inflammatory response could lead to a chronic phase of the disease, known as long COVID, affecting the whole body [23].

A relevant piece of data worth mentioning is related to hospitalized patients who ended up developing neurological manifestations after SARS-CoV-2 infection of early and acute origin: when observing the profile of patients involved, the vast majority were elderly, obese, with dyslipidemia, and with diabetes and hypertension. Between post-COVID patients, a disproportional increase was seen in the prevalence of neuropsychiatric injury, myopathies, strokes, status epilepticus, and encephalitis when comparing to published studies on pre-existing neurological disorders. Chronification, however, may or may not be associated with increased mortality in infected patients [24].

When analyzing patients with post-COVID sequelae, the following consequences are observed: homonymous hemianopia, speech disorders, reduced level of consciousness, aphasia, and motor incoordination, among others [25].

In the cardiovascular system, the main pathway for SARS-CoV-2 cell entry is the ACE-2 receptor too [17]. This receptor is highly present in myocardial cells [17,18]. In this sense, the cardiac tissue is in elevated risk for cellular injury. In addition, the ACE-2 activity is increased in patients with underlying cardiovascular disease, and this could contribute to a more severe course of the disease in this population [18].

### 3.2. Acute Complications

#### 3.2.1. Acute Complications Related to Neurological System

As previously mentioned, the mechanism by which COVID-19 acts on the neurological system is not fully understood; therefore, it is still unclear whether the neurological manifestations are just an epiphenomenon of a respiratory disease or if they are directly related to the infection by SARS-CoV-2 [26,27].

Early manifestations include olfactory and gustatory dysfunction, peripheral nervous system and cranial nerve pathologies, signs of corticospinal tract dysfunction, cognitive changes, delirium, seizures, meningitis, encephalitis, myelitis, and acute cerebrova50cular disease [26,28]. In this context, it is noteworthy that, when comparing to acute respiratory distress syndromes caused by other infectious agents, patients hospitalized for COVID-19 had a significantly higher risk of developing neurological complications (RR = 1.98; 95%CI 1.23–3.26)—even when directly assessing corticospinal tract dysfunctions, which are also more prevalent in this group (RR = 1.62; 95%CI 0.72–3.44) [29].

##### Anosmia and Ageusia

The most frequently reported early and late neurological complications are summarized in Table 1. Regarding the impairment of smell and taste, it was noted that anosmia persisted among patients, on average, for 11 days (ranging from 5 to 25 days), while ageusia lasted for an average of 8 days (ranging from 4 to 25 days) [30]. Furthermore, these symptoms are more present in women and people under the age of 51 years [31]. Although olfactory dysfunction is common in other viral infections, due to mucosal inflammation, what appears to be unique in COVID-19 is the development of anosmia/hyposmia in the absence of nasal obstruction or rhinorrhea, due to the tropism of SARS-CoV-2 for the olfactory nerve and the presence of ACE-2 receptors in the nasal and oral mucosa of the tongue [32].

Giacomelli et al. applied a simple questionnaire to 88 hospitalized patients with SARS-CoV-2 infection and 33.9% of them reported olfactory or gustatory disturbances, while both were subjectively detected at the same time in almost 20% of them [33]. Regarding the time of detection of these symptoms, 20.3% and 91% of the patients reported, respectively, anosmia and ageusia before hospitalization. This study also observed that olfactory and/or gustatory disorders were more frequent in female and young patients [33].

##### Acute Cerebrovascular Disease

COVID-19 patients are at risk for cerebrovascular complications, especially when having additional comorbidities. The SARS-CoV-2 virus specifically binds to angiotensin-converting enzyme 2 (ACE-2) receptors, which is able to compromise the blood pressure regulation system, especially in hypertensive patients [36]. Between coagulopathy patients, COVID-19 may increase the risk for cerebral venous sinus thrombosis and cerebral hemorrhage. The most common manifestations associated with these complications are an altered level of consciousness, mental confusion, ataxia, headache, convulsion, plegia, and paresis [37].

**Figure 2:**
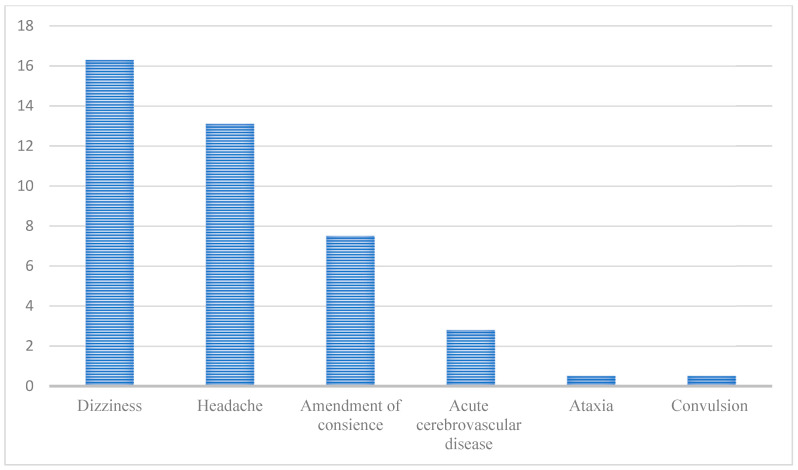
Neurological manifestations in patients with COVID-19 based on a Chinese study by Maury et al. [34]. (Reference: elaborated by the authors).

In one study with 214 COVID-19 hospitalized patients conducted in Wuhan, China, 78 (36.4%) had manifestations associated with the nervous system. The involvement of the central nervous system, especially, was observed in 53 patients (24.8%), including dizziness (*n* = 36; 16.8%), headache (*n* = 28; 13.1%), consciousness-level alterations (*n* = 16; 7.5%), acute cerebrovascular disease (*n* = 6; 2.8%), ataxia (*n* = 1; 0.5%), and convulsion (*n* = 1; 0.5%), according to what is elucidated in Figure 2 below [34].

A survey carried out in the United Kingdom, with 125 COVID-19 patients, noticed that cerebrovascular events were present in 77 (62%), of which stroke was the main associated complication, with 57 (74%) cases [38].

Moreover, the blockade of angiotensin II in the renin-angiotensin system contributes to difficulties in the expression of angiotensin I and add up to endothelial dysfunction, damages organs, and consequently, increases the chance of stroke [38]. Therefore, in general, treatments with antagonists of the renin-angiotensin system generate positive impacts in patients who suffer from stroke, in concomitance to COVID-19 [39]. 

Furthermore, elevated levels of D-dimer and fibrinogen are alarm signs in patients with severe COVID-19. These are serum markers for a condition called sepsis-induced coagulopathy [39]. In this context, the stroke risk between individuals infected with SARS-CoV-2 increases when facing hypoxia, inflammation, and diffuse intravascular coagulation mechanisms for thromboembolic disease [40].

#### 3.2.2. Acute Complications Related to Cardiovascular System

In the retrospective observational study conducted by Kunal et al., with 108 patients that were hospitalized for COVID-19, acute cardiac injury was the most common cardiovascular complication, being reported in 28 (25.9%) patients, followed by heart failure (HF), cardiogenic shock (CS), and acute coronary syndrome (ACS) in 4 (2.8%) patients each, and pericardial effusion reported in 2 (1.9%) subjects [41].

##### Myocardial Injury and Myocarditis

The pathogenesis related to myocardial injury in COVID-19 is still unknown, but it can be related to a hyperimmune response, or maybe to the increased expression of ACE-2 receptors in myocardial cells [42,43]. The occurrence of myocardial injury appears to be independent of preexisting cardiovascular risks [42].

Myocardial injury, as indicated by elevated troponin T (TnT) levels, is significantly associated with fatal outcome in COVID-19. In a retrospective observational study, conducted with 187 patients in Wuhan, China, 52 (27.8%) exhibited myocardial injury during COVID-19 hospitalization. Between patients without underlying cardiovascular disease, mortality for those with normal TnT levels was 7.26% (8 out of 105), and for the ones with elevated levels, the mortality rate was 37.5% (6 out of 16). For the patients with underlying cardiovascular disease and normal TnT levels, mortality was 13.33% (4 out of 30); for those with cardiovascular disease and elevated TnT levels, the rate was 69.44% (25 out of 36) [44].

Although troponin serum levels are commonly elevated during COVID-19 hospitalization, differentiation between myocarditis and other causes, such as stress cardiomyopathy and myocardium infarction, may be challenging [45]. Myocarditis may be suspected when troponin significantly rises in the absence of myocardial ischemia. However, second-level testing (e.g., cardiovascular magnetic resonance) for COVID-19-suspected myocarditis often has a limited availability, making a final diagnosis difficult [42]. 

Transthoracic echocardiography is the first-line imaging test performed for COVID-19-related myocarditis. The major findings in this test are global and regional hypokinesia, as well as an increase in wall thickness [42].

In a meta-analysis including 215 patients with confirmed COVID-10-related myocarditis, cardiac markers were reported in 212 of those, of which 201 (94.8%) had elevated levels. Electrocardiography findings were obtained in 96 of these patients: 2 (2%) had normal findings, whereas 43 (44.8%) had ST segment elevation, and the rest had many other unspecific alterations. In this same meta-analysis, echocardiography was conducted in 175 patients: 55 (31.4%) demonstrated a reduced ejection fraction, 12 (6.9%) had pleural effusion, 7 (4%) had left-ventricular hypertrophy, and myocardial dyskinesia was seen in 19 (10.9%) of them; 45 of the 215 underwent cardiovascular magnetic resonance, and 36 of them were diagnosed with myocarditis by the Lake Louis criteria [43].

##### Stress Cardiomyopathy

Stress cardiomyopathy (Takotsubo cardiomyopathy, or “broken heart syndrome”) is characterized by left ventricular dysfunction, usually caused by high catecholamine elevations, in the setting of physical or emotional stress [46].

Since COVID-19 is associated with a systematic cytokine storm, stress cardiomyopathy may be a dangerous complication for this disease. A systematic review of 12 published cases, from 2020, revealed that the most common symptoms of COVID-19-related stress cardiomyopathy were dyspnea (100%), fever (66.6%), and chest pain (58.3%). Nine of the reported cases had heart failure with reduced ejection fraction on echocardiography. The mean left ventricular ejection fraction (LVEF) was 40.6 ± 9.9%. Out of 12 cases, 11 (91.6%) had at least one complication, six developed cardiac complications with one case each of cardiac tamponade, heart failure, myocarditis, hypertensive crisis, and cardiogenic shock in two [46].

A systematic review of comparative studies, between 52 patients with COVID-19-related Takotsubo cardiomyopathy (TCM), COVID-19 patients with other causes of myocardial injury, and COVID-19 patients without myocardial injury, found that those with TCM had higher mortality (40.0%) than those with other myocardial injuries (30.0%) and those without myocardial injury (2.3%) [47].

##### Arrhythmias

Electrocardiography (ECG) is a broadly available diagnostic test that could be very important in the detection of the life-threatening cardiovascular complications of COVID-19. The arrhythmia types related to COVID-19 seem to be unspecific and variable. The common ECG findings in the observational study by Kunal et al. were sinus tachycardia in 18 (16.9%), followed by first-degree atrioventricular (AV) block in 5 (4.6%), ventricular tachycardia/ventricular fibrillation in 2 (1.8%), and sinus bradycardia in 1 (0.9%) [41]. Another observational study, by Mccullough et al., with 756 COVID-hospitalized patients who underwent ECG tests, reported that the most common conduction abnormalities were nonspecific repolarization abnormalities in 29.0%, localized T-wave inversion in 10.5%, right bundle branch block in 7.8%, atrial premature contractions (APC) in 7.7%, atrial fibrillation/flutter in 5.6%, and first-degree AV block in 2.5% [48].

In a meta-analysis by Pellicori et al., the weighted mean incidence (WMI) for supraventricular arrhythmias was 8.5%, for ventricular arrhythmias it was 2.7%, and for either or otherwise unspecific arrhythmias the WMI was 9.3%. These arrhythmias were more likely in critically ill patients, those with elevated troponin levels, and patients who received therapies that are known to prolong the QT interval, such as hydroxychloroquine, especially when given in combination with azithromycin [49].

The arrhythmias seen in COVID-19 may also have an important prognostic value in this disease. The study by Mccullough revealed that APCs, right bundle branch block, localized T-wave inversion, and nonspecific repolarization abnormalities were associated with increased odds of death after accounting for age and other important clinical characteristics [48].

##### Heart Failure

Research developed in Madrid, Spain, using data from the hospital’s emergency department from 1 March to 20 April in 2020, aimed to study confirmed cases of COVID-19 infection and its relation to chronic heart failure (CHF) and acute heart failure (AHF). The investigation considered CHR as any patient with a previous congestive decompensation or left ventricular systolic ejection fraction lower than 40%. AHF was defined as a fast aggravation of the symptoms or their beginning, and/or signs of heart failure during the time of the research [50].

In the study, 3080 patients with confirmed SARS-CoV-2, by RNA reverse-transcriptase polymerase chain reaction (RT-PCR), were analyzed, of which, 152 had chronic heart failure. Based on the outcomes of each one, the study concluded that the majority of AHF cases occurred on patients without previous cases of heart failure (77.9%), although the proportion of patients who progressed to AHF was bigger in the chronic heart-failure group (11.2% vs. 1.8%). The mechanism of this interaction is still not completely clear, but there are some hypotheses that corelate heart failure and COVID-19 infection because of its severe proinflammatory cytokine-modulated reaction, endothelial dysfunction, hypercoagulable state, and plaque rupture [50].

### 3.3. Long-Term Complications

#### 3.3.1. Neurologic Long-Term Complications

With regard to neurological complications, although SARS-CoV-2 infections generally have systemic and, mainly, respiratory repercussions, a study carried out in Wuhan, China, was the first to point out that neurological manifestations also deserve to be highlighted. Neurological long-term complications were revealed to be present in 36% of hospitalized patients [34]. Thus, it is known that clinical manifestations may be present in the beginning of the infection but also as long-term sequelae, including memory loss, attention deficit, and lower processing speed are possible complications [35]. Regarding memory loss, it is worth mentioning that more than 30% of post-COVID-19 individuals report this symptom [51]. In addition, COVID-19 is known to cause a “cytokine storm”, and, in turn, IL-4, involved in brain function (such as memory) and in the beneficial and counteractive role of pro-inflammatory cytokines, remained significantly elevated in all those affected by COVID-19 [52]. Its elevation may signal a response to the threat of neuroinflammation and an attempt to restore homeostasis [53].

One study suggests that different profiles of serum and CSF cytokines are associated with different manifestations [54]. For example, encephalopathy has been associated with increased serum IL-6 levels, a predictor of poor prognosis. CSF IL-2 levels were also elevated in these patients. Thus, encephalopathy appears to be multifactorial in origin, including immune-mediated processes. Infections associated with COVID-19, however, have been associated with high serum levels of IL-6, CXCL8, and CXCL10 [54]. The release of these pro-inflammatory substances increases the permeability of the blood–brain barrier, which, in turn, promotes the transport of cytokines to the CNS, leading to microglial and astrocytic activation. Activated microglia release other inflammatory mediators, including interleukins, complement factors, TNF-ÿ, glutamate, and quinolinic acid. This leads to increased levels of glutamate and NMDA receptor expression, resulting in excitotoxicity and neuronal loss, which can cause memory and learning problems, hallucinations, and neuroplasticity changes. Indeed, microglial and astroglial changes are similar to those seen in several neurodegenerative diseases; these patients also have impaired synaptic transmission in neurons involved in cognitive function, which may at least partially explain the neurological manifestations of the infection [16].

When looking at the inflammatory context in the body caused by cytokines released on a large scale, there is significant evidence that the appearance of vascular complications is more common because of the consequent hyperactivation of inflammatory factors and dysfunction of the coagulation system—especially D-dimer and abnormalities platelets—which increases the risk of cerebrovascular disease [36].

One study indicated that even small differences in the severity of the infectious condition can interfere with long-term manifestations, a conclusion based on previous analyses, consultations, and complete neurological examinations after 6 months of hospitalization. This severity/symptoms relationship was more evident for cognitive symptoms, such as memory complaints and attention deficit, while, for example, myalgia and numbness seemed to be independent of severity [55]. In this sense, there is a possibility that the central nervous system is more vulnerable to severe SARS-CoV-2 infection, possibly through a higher general inflammatory response and longer hospitalization—as is already known to occur in other infectious diseases [56].

#### 3.3.2. Cardiovascular Long-Term Complications

Complications are frequently observed in patients recovering from COVID-19, ranging from mild to life-threatening conditions. Follow-up and multi-disciplinary rehabilitation are needed for patients who presented arrhythmias, myocarditis, or other cardiac complications during the acute phase of the disease [57]. Long-term cardiovascular damage after COVID-19 infection can happen because of a variety of intercalated causes such as viral persistence, immune dysregulation, and autoimmunity [58].

Data regarding the cardiovascular manifestations of long COVID syndrome are still very scarce. An observational study, with 100 patients who recovered from COVID-19 infection and underwent a cardiovascular magnetic resonance, in a median of 71 days post-COVID-19 diagnosis, revealed that 78 (78%) patients had cardiovascular involvement. The most common abnormality was myocardial inflammation (defined as abnormal native T1 and T2 measures), detected in 60 patients, followed by regional scar and pericardial enhancement [59].

To summarize the main acute and late cardiovascular complications related to COVID-19, they are reported in Table 2.

### 3.4. Post-Vaccine Complications

#### 3.4.1. Cardiovascular Post-Vaccine Complications

The COVID-19 pandemic has caused tragic effects on public health and over 600 million deaths [60,61]. In the meanwhile, vaccination was developed as an effort to reduce the spread of this virus, and it has really shown its efficiency as a strategy to slow down the pandemic [60,61]. However, with the widespread vaccine application, some adverse effects in various organ systems were reported, such as cardiovascular complications [61].

**Figure 3:**
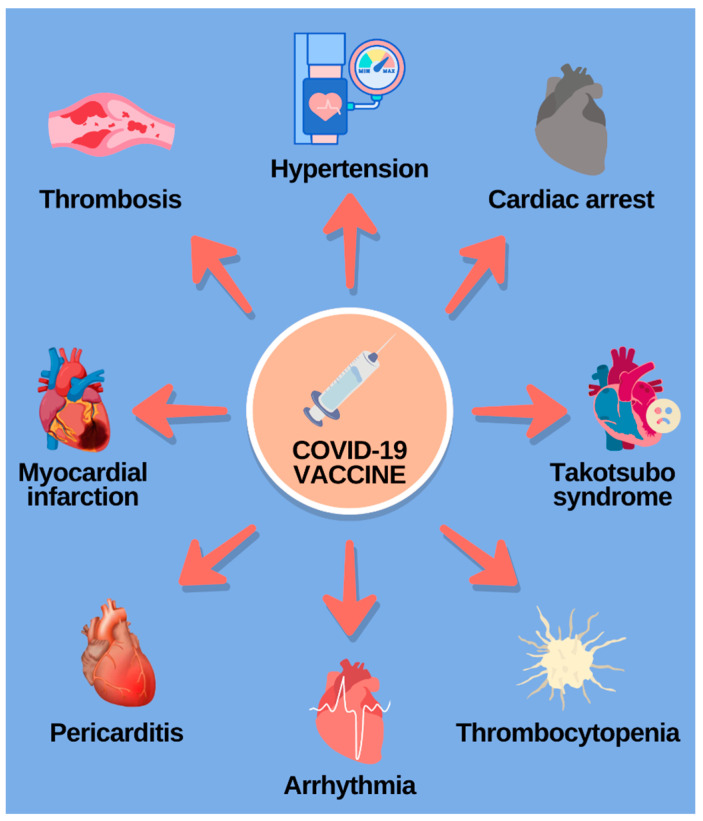
Diverse cardiovascular complications following the COVID-19 vaccination. (Reference: elaborated by the authors, based on Liu, R.; Pan, J.; Zhang, C.; Sun, X. Cardiovascular Complications of COVID-19 Vaccines. *Front Cardiovasc Med.* **2022**, *9*, 1–7 [60]).

Some of the cardiovascular adverse effects following the vaccination are myocarditis and pericarditis, thrombosis, thrombocytopenia, hypertension, acute coronary syndrome, arrythmias, Takotsubo cardiomyopathy, and cardiac arrest and death [60,61], and they are summed up in Figure 3. Some of these manifestations will be elucidated in the text below.

From 29 December 2020 to 11 June 2021, there were 1226 notifications of myocarditis after mRNA vaccination on the vaccine adverse event reporting system (VAERS) [60]. Myocarditis and pericarditis both have as clinical signs chest pain, tachypnea, palpitations, headache, fever, and cough. In addition, it has been reported that these pathologies occur the most within some days after the immunization, and they are more common after the second dose [61], although the mechanisms of the mRNA vaccine responsible for causing those adverse effects are not completely uncovered [60,61].

In addition, the pathophysiology of thrombosis after vaccination is not elucidated either, but there is a hypothesis that it is associated with the encoding of the SARS-CoV-2 spike protein by the mRNA vaccine. This protein induces the activation of endothelial brain cells, pro-inflammatory cytokines, and intercellular adhesion molecule 1, as well as promoting platelet aggregation and the exudation of dense granules from the thrombocytes. The causing mechanisms of vaccine-induced thrombocytopenia are not clear either, but it is known that vaccines can trigger the immune system to produce antiplatelet autoantibodies through molecular mimicry and autoimmunity, and that can generate thrombocytopenia [60].

The supposed mechanism of the vaccine-induced thrombosis can also be responsible for myocardial infarction (MI) and coronary disease after COVID-19 vaccination, but the data about MI related to the vaccine are still inconclusive. It has been identified that MI is more common after the first dose of vaccination, and it usually occurs after a period that variates from 15 min to 2 days. Besides that, studies have proven that the risk of having a myocardial infarction with COVID-19 in unvaccinated people is higher than the risk of having the same abnormality because of the vaccination [61].

#### 3.4.2. Neurologic Post-Vaccine Complications

In addition to the self-limited complications common to different types of vaccine (such as pain at the application site, weakness, rash, fever/chills, myositis, and headache) and the cardiovascular complications mentioned above, neurological complications have been reported in the literature, in particular the Guillan–Barré syndrome.

The Guillain–Barré syndrome is an acute immune polyradiculoneuropathy characterized by involvement of motor, sensory, and autonomic nerves that, usually, is related to a fast and progressive flaccidity, weakness, and paresthesia of the limbs distally [62]. That syndrome tends to be a significant concern as a serious possibility of adverse effects to the vaccine. Regarding its pathophysiology, there is a hypothesis that an immune response after vaccination may produce an autoimmune process and, consequently, lead to the production of autoantibodies against myelin [63].

As mentioned above, there have been documented various neurological and cardiovascular comorbidities probably related to COVID-19 vaccination (Table 3). Besides that, the pathophysiology of most of these interactions are not completely elucidated, and those effects are still uncommon considering the vast number of people all over the world who have been vaccinated [61].

## 4. Conclusions

This article summarizes and clarifies some of the main cardiovascular and neurological short/long-term and post-vaccination manifestations associated with SARS-CoV-2 infection, some of which are still unknown by a significant number of non-specialist physicians in the field. Based on this understanding, the medical service becomes more aware of the causal relationship between some conditions and COVID-19, ergo, being more prepared for the most prevalent complications, to associate and, consequently, to treat patients earlier. Therefore, there is a chance of better prognoses in this context and the need to increase the number of studies about complications related to SARS-CoV-2 infection for a better understanding of other associated conditions.

## Figures and Tables

**Table 1:** Immediate and late neurological complications reported in COVID-19 studies. (REFERENCE: elaborated by the authors, based on Stiller et al. [26], Madani Neishaboori [28], Boscutti et al. [33], Maury et al. [34], and Fotuhi et al. [35].

Neurological Complications	
Early Complications	Late Complications
Anosmia/ageusia	Memory problems
Acute cerebrovascular disease	Learning problems
Delirium	Hallucinations
Seizures	Alteration of neuroplasticity
Encephalopathy	Increased risk of stroke

**Table 2:** Immediate and late cardiovascular complications reported in COVID-19 studies. (Reference: elaborated by the authors, based on Kunal et al. [41], Castiello et al. [42], Singh et al. [46], Mccullough et al. [48], Rey et al. [50], and Puntmann et al. [59].).

Cardiovascular Complications	
Early Complications	Late Complications
Myocardial injury and Myocarditis	Myocardial inflammation
Stress cardiomyopathy	Regional scar
Arrhythmias	Pericardial enhancement
Heart failure	

**Table 3:** Post-vaccine cardiovascular complications of COVID-19.

Manifestation	Total Cases N	Janssen N (%)	Moderna N (%)	Pfizer N (%)	Unknown N (%)
Acute cardiac event	68	4 (5.8%)	22 (32.3%)	42 (61.7%)	-
Arrhythmia	16,046	475 (2.9%)	2606 (16.2%)	12,881 (80.2%)	84 (0.5%)
Cardiac failure	3325	88 (2.6%)	650 (19.5%)	2562 (77.0%)	25 (0.7%)
Cardiac valve disease	137	10 (7.2%)	26 (18.9%)	100 (72.9%)	1 (0.7%)
Cardiomegaly	1315	61 (4.6%)	437 (33.2%)	808 (61.4%)	9 (0.6%)
Endocarditis	234	14 (5.9%)	50 (21.3%)	170 (72.6%)	-
Myocarditis	16,902	255 (1.5%)	3929 (23.2%)	12,641 (74.7%)	77 (0.4%)
Pericarditis	11,241	204 (1.8%)	2108 (18.7%)	8866 (78.8%)	63 (0.5%)

Source 1—United States Department of Health and Human Services (DHHS), Public Health Service (PHS), Centers for Disease Control (CDC)/Food and Drug Administration (FDA), vaccine adverse event reporting system (VAERS) 1990–17 February 2023, CDC WONDER On-line Database. Accessed at http://wonder.cdc.goc/vaers.html on 27 February 2023 6:50:20 P.M.
